# Risk factors of hepatic dysfunction in patients with Graves’ hyperthyroidism and the efficacy of ^131^iodine treatment

**DOI:** 10.1097/MD.0000000000006035

**Published:** 2017-02-03

**Authors:** Renfei Wang, Jian Tan, Guizhi Zhang, Wei Zheng, Chengxia Li

**Affiliations:** Tianjin Medical University General Hospital, Department of Nuclear Medicine, Heping, Tianjin, China.

**Keywords:** ^131^i treatment, efficacy, Graves hyperthyroidism, hepatic dysfunction, risk factors

## Abstract

Hepatic dysfunction is often observed in patients with Graves’ hyperthyroidism. The aims of this study were to investigate the risk factors for hepatic dysfunction and to analyze the efficacy of ^131^I (radioactive iodine-131) treatment. In total, 2385 patients with Graves’ hyperthyroidism (478 males, 1907 females; age 42.8 ± 13.5 years) were involved in our study. Of these, 1552 cases with hepatic dysfunction received ^131^I treatment. All clinical data were retrospectively reviewed to explore the risk factors associated with hepatic dysfunction using logistic regression analysis. Furthermore, we observed thyroid and liver function indices for the 1552 subjects at 3, 6 and 12 months after ^131^I treatment, in order to evaluate efficacy. Overall, 65% patients were affected by hepatic dysfunction. The most common abnormality was elevated alkaline phosphatase (ALP), of which the prevalence was 52.3%. The percentages of hepatocellular injury type, bile stasis, and mixed type were 45.8%, 32.4%, and 21.8%, respectively. Both univariate and multivariate analyses demonstrated that age, duration of Graves hyperthyroidism, free triiodothyronine (FT_3_)level, and thyrotrophin receptor antibody (TRAb) concentration were the most significant risk factors predicting hepatic dysfunction. Additionally, the patients with mild hepatic dysfunction, or hepatocellular injury type were more likely to attain normal liver function after ^131^I treatment. Furthermore, after ^131^I treatment, liver function was more likely to return to normal in the cured group of patients compared with the uncured group. Older patients and cases with a longer history of Graves’ hyperthyroidism, higher FT_3_ or TRAb concentration were more likely to be associated with hepatic dysfunction, and the prognosis of hepatic dysfunction was closely associated with the outcomes of Graves’ hyperthyroidism after ^131^I treatment.

## 1 Introduction

Graves hyperthyroidism is an autoimmune thyroid disease which can affect multiple organ systems, including the cardiovascular, gastrointestinal, and hepatic systems. In clinical practice, hepatic dysfunction in patients with Graves hyperthyroidism is common.^[1,2]^ Although most patients have no obvious clinical symptoms, except for abnormal liver function indices, serious liver injury, and even liver failure can occur in a few patients.^[3–5]^

There are 3 main factors that contribute to hepatic dysfunction in the context of hyperthyroidism, including the independent effects of excessive thyroid hormones, antithyroid drug (ATD)-related liver injury, and the presence of concomitant liver disease. Historically, the control of hyperthyroidism has required 3 options including ATD, surgery, and radioiodine-131 therapy (RIT). In China, the current treatment guideline for Graves hyperthyroidism states that RIT should be the first choice of treatment in patients with hepatic dysfunction.^[6]^ This has proven to be a relatively safe procedure with a high cost-effective ratio in retrospective studies. The normal interaction between the thyroid and liver is critical for maintaining homeostasis. However, to date, reports demonstrating predictive factors related to hepatic dysfunction in Graves’ hyperthyroidism are both scarce and controversial, and studies exploring RIT and liver function are rare. Therefore, identification of the factors resulting in hepatic dysfunction is crucial.

In the present study, we retrospectively collected large-scale clinical data on 2385 patients with Graves hyperthyroidism to investigate the risk factors for hepatic dysfunction in patients with Graves hyperthyroidism and further analyzed the therapeutic effect of ^131^I treatment.

## 2 Materials and methods

### 2.1 Ethics statement

This study is a retrospective clinical study. It is the summary and analysis of a large number of clinical data. The ethics committee of Tianjin Medical University General Hospital waived the need to obtain written informed consent from all patients. All clinical data used in this study were analyzed anonymously.

### 2.2 Patients

A total of 2385 patients who were diagnosed with Graves hyperthyroidism (478 males, 1907 females; age 42.8 ± 13.5 years) were retrospectively reviewed. Graves hyperthyroidism was diagnosed on the basis of thyrotoxicosis, increased thyroid hormones and decreased thyroid-stimulating hormone (TSH), elevated thyroid radioiodine uptake, and diffuse goiter, or exophthalmos, or pretibial myxedema, or positive TRAb.^[7]^ Of these, 1073 patients had been treated with ATD before, and 1552 cases were associated with hepatic dysfunction and received RIT. We used hepatitis virus markers, abdominal ultrasonography, echocardiography, and determination of auto-antibodies and immunoglobulin subtypes for patients with hepatic dysfunction, in order to exclude other apparent causes of liver damage. Other causes included viral hepatitis, liver cirrhosis or biliary tract disease, chronic cardiac dysfunction or autoimmune liver disease.

### 2.3 Data collection and grouping

Data were recorded, including patient age (named X1), gender (X2), duration of Graves hyperthyroidism (X3), the course of ATD treatment (X4), heart rate (X5), serum free triiodothyronine (FT_3_) (X6), free thyroxine (FT_4_) (X7), TSH (X8), TRAb (X9), anti-thyroid peroxidase antibody (TPOAb) (X10) and anti-thyroglobulin antibody (TgAb) (X11), RAIUmax (maximum radioiodine uptake) (X12), and thyroid weight (X13). Meanwhile, liver function tests including serum aspartate aminotransferase (AST), alanine aminotransferase (ALT), ALP, gammaglutamyl transpeptidase (GGT), total bilirubin (TBIL), and direct bilirubin (DBIL) were also collected.

The diagnosis of hepatic dysfunction was based on the following criteria:^[8]^ ALT, AST or GGT < 3 times the upper limit of normal (ULN), ALP < 2 ULN and/or TBIL, DBIL < 2.5 ULN was defined as mild hepatic dysfunction; 3 ULN≤ ALT or AST < 20 ULN, 3 ULN≤ GGT < 10 ULN, 2 ULN≤ ALP < 5 ULN and/or 2.5 ULN≤ TBIL, DBIL < 5 ULN as moderate; ALT or AST ≥20 ULN, GGT ≥10 ULN, ALP ≥5 ULN and/or TBIL, DBIL≥5 ULN as severe.

According to the different liver function indices, hepatic dysfunction was further classified as hepatocellular injury type, bile stasis type, or a mixed type.

### 2.4 Parameter assessments

Thyroid function tests were measured by chemiluminescence immunoassays (ADVIA CENTAUR XP SIEMENS AG). TPOAb and TgAb were detected by immulite (Immulite 2000 SIEMENS AG), and TRAb was detected by ELISA (enzyme-linked immunosorbent assay) (MEDIPAN Germany). Liver function indexes were measured by colorimetry (Hitachi C7600 Japan).

Length, breadth, and depth of the 2 lobes of the thyroid gland were measured by ultrasound using the formula for a prolate ellipsoid.^[9]^ The ^131^I dose (MBq) = estimated thyroid weight (g) × absorption dose (Gy/g) × 0.67/ [effective half-life (EHL), (days) × RAIUmax].^[10]^ Absorption dose = 100 Gy/g and 0.67 is a correction coefficient. The dosage range of ^131^I was 74–555 MBq (240.5 ± 81.4 MBq).

### 2.5 Patient follow-up and efficacy after^131^I treatment

We measured serum levels of thyroid and liver function indices on the 1552 patients at 1, 3, 6, and 12 months after ^131^I therapy to evaluate the treatment efficacy. We defined complete remission (euthyroid) and hypothyroidism as “cure” (cured group), and partial remission, inefficacy, or recurrence as “uncure” (uncured group).

### 2.6 Statistical analysis

A chi square test was used to analyze the difference between the ratios. To identify the risk factors for hepatic dysfunction, we used a bivariate logistic regression model (univariate analysis) and stepwise logistic regression (multivariate analysis) with a variable entrance criterion of 0.05 or less. Missing values for TgAb and TPOAb were assigned to the mean level. All *P* values presented were 2-tailed, and values < 0.05 were considered to be statistically significant. Statistical analysis was performed using SPSS (Statistical Package for Social Sciences) for windows, version 12.0 (SPSS, Chicago, IL).

## 3 Results

### 3.1 Clinical features of hepatic dysfunction

Overall, 65% (1552/2385) of patients with Graves hyperthyroidism were affected by hepatic dysfunction. Moreover, we found the prevalence of hepatic dysfunction was 68.1% (731/1073) in ATD-treated patients, and prevalence was 62.6% (821/1312) in patients without ATD treatment. The patients who received ATD treatment had a significantly higher prevalence of hepatic dysfunction compared with those who did not receive ATD treatment (*P* = 0.005).

Most patients with hepatic dysfunction had no obvious clinical symptoms except for abnormal liver function indices. The prevalence of mild, moderate, and severe hepatic dysfunction were 58.5% (908/1552), 34.9% (542/1552), and 6.6% (102/1552), respectively. The most common abnormality was elevated ALP, with a prevalence of 52.3%. Additionally, the percentages of hepatocellular injury type, bile stasis, and mixed type were 45.8%, 32.4%, and 21.8%, respectively.

### 3.2 Risk factors for hepatic dysfunction in Graves hyperthyroidism

Patient characteristics were compared using bivariate logistic regression analysis between the 2 groups (Table 1). The results showed patients aged ≥45, duration of Graves hyperthyroidism >3 years, heart rate >90 beats per minute (bpm), FT_3_ level >19.5 pmol/L (3 ULN), TRAb concentration >15 IU/L (10 ULN), and positive TPOAb were more likely to be associated with hepatic dysfunction (OR: 1.453–3.985, all *P* < 0.01).

**Table 1:**
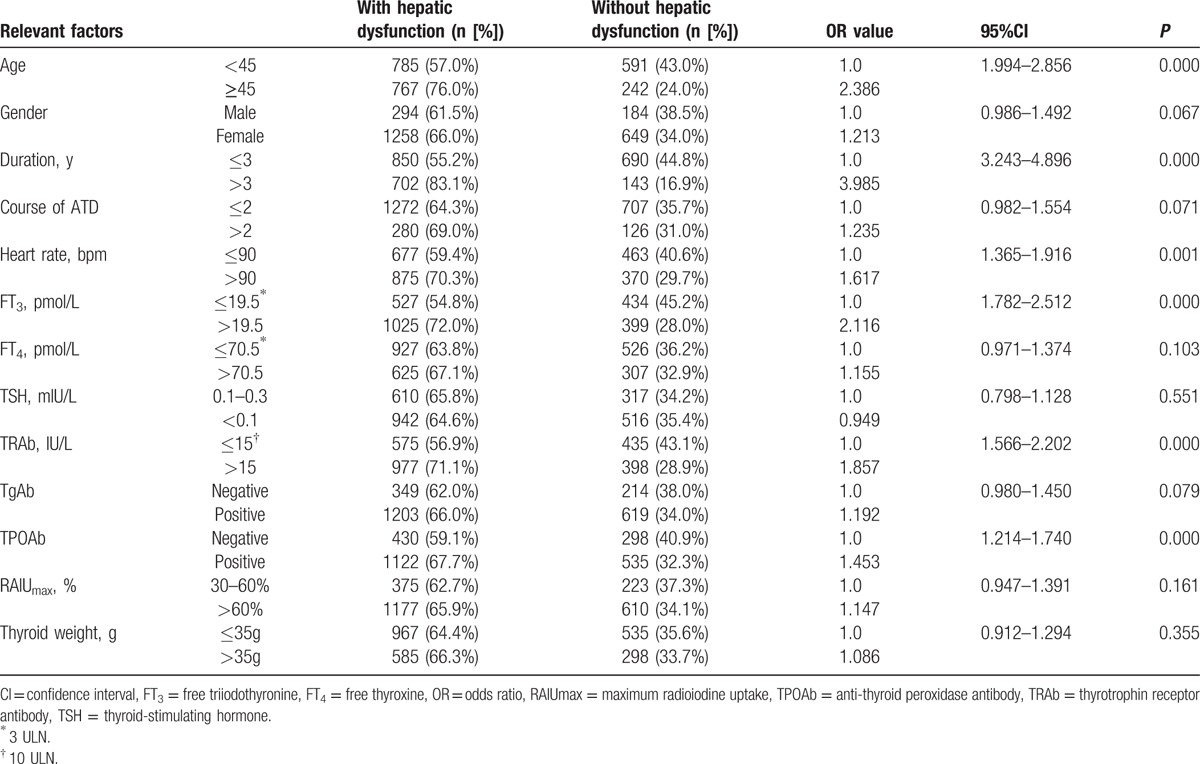
Bivariate logistic regression analysis of the factors for Graves’ hyperthyroidism accompanied by hepatic dysfunction.

Furthermore, we used multivariate logistic regression analysis to screen for relevant factors. In our study, we suggested a new assignment for causal variables: X_1_ = age/10; X_2_ = 1 if the patient was male, and X_2_ = 2 if the patient was female; X_3_ = 1, 2, 3, 4, 5, if the duration of hyperthyroidism was less than 1 year, 1–2 years, 2–3 years, 3–4 years and longer than 4 years, respectively; X_4_ = 1, 2, 3, 4, 5, if the patient did not receive ATD treatment, or accepted drug therapy that was shorter than 1 year, 1–2 years, 2–3 years, and longer than 3 years; X_5_ = 1, 2, 3, 4, if the heart rate was lower than 80, 80–90, 90–100, and exceeded 100bpm; X_6_ = 1, 2, 3, 4, if FT_3_ level was lower than twice the upper limit, 2–3, 3–5, and higher than 5 times the upper limit; X_7_ = 1, 2, 3, 4, if FT_4_ level was lower than twice, 2–3, 3–5, and higher than 5 times the upper limit; X_8_ = 1 if TSH was 0.1–0.3 mIU/L, and X_8_ = 2 if it was lower than 0.1 mIU/L; X_9_ = 1, 2, 3, 4, if TRAb was normal, 1.5 (upper limit)-15, 15–40 (determination limit), >40 IU/L; X_10_ = 1, 2, 3, if TgAb was negative, 40 (upper limit)-3000 (determination limit), >3000 IU/mL; X_11_ = 1, 2, 3, if TPOAb was negative, 35 (upper limit) – 1000 (determination limit), >1000IU/ml; X_12_ = RAIUmax (%) × 10; X_13_ = thyroid weight (g)/10. Resultant variable: Y = 1 for the patient with hepatic dysfunction, and Y = 0 for the patient without hepatic dysfunction. Forward stepwise regression analysis rejecting for trend ultimately revealed that age, duration of Graves hyperthyroidism, FT_3_ level and TRAb concentration were independent factors predicting hepatic dysfunction in patients with Graves hyperthyroidism (Table 2). Regression equation: Y = 1.556X_1_+2.342X_3_+0.985X_6_+0.577X_9_−2.217 (likelihood ratio test, *P* < 0.01).

**Table 2:**
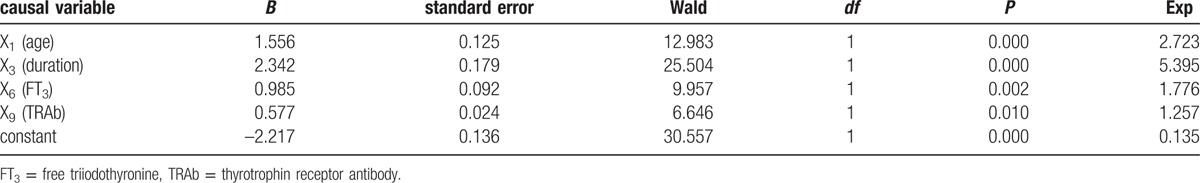
Variables and constants of the regression equation.

### 3.3 Outcome of hepatic dysfunction after ^131^I treatment

The outcome of hepatic dysfunction after ^131^I treatment is displayed in Table 3. In total, the liver function tests returned to normal in 77.3% (1200/1552) of patients with hepatic dysfunction 6 months after ^131^I treatment. Moreover, we found that the remission rates in patients with mild, moderate, and severe hepatic dysfunction were 86.8%, 65.5% and 55.9%, respectively. Additionally, the remission rate of mild hepatic dysfunction was higher than that of moderate (*P* < 0.001) or severe dysfunction (*P* < 0.001). Similarly, we found the remission rates with hepatocellular injury type, bile stasis, and mixed type were 91.7%, 59.2%, and 74.0%, respectively, and the remission rate of hepatocellular injury type was also higher than that of bile stasis (*P* < 0.001) or the mixed type (*P* < 0.001).

**Table 3:**
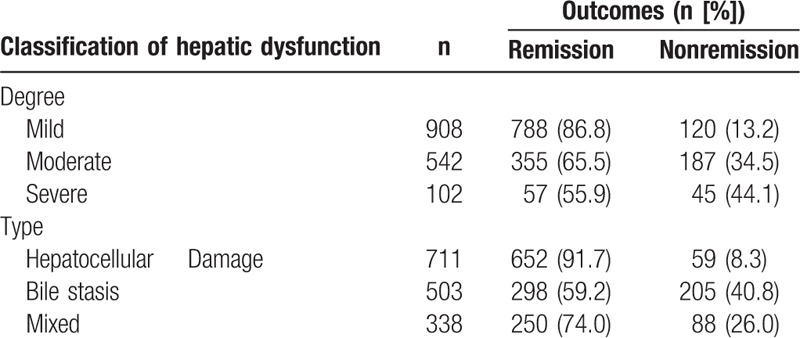
Outcomes of hepatic dysfunction of different degrees and types after ^131^I treatment.

### 3.4 Efficacy of Graves’ hyperthyroidism and outcome of hepatic dysfunction after ^131^I treatment

We measured serum levels of thyroid and liver function indices on the 1552 patients with Graves’ hyperthyroidism at 3, 6, and 12 months after ^131^I treatment to evaluate the therapeutic effect. In the present study, we defined complete remission (euthyroid) and hypothyroidism as cure (cured group), and partial remission, inefficacy or recurrence as uncure (uncured group). The relationship between efficacy of Graves’ hyperthyroidism and outcomes of hepatic dysfunction after ^131^I treatment are displayed in Table 4.

**Table 4:**
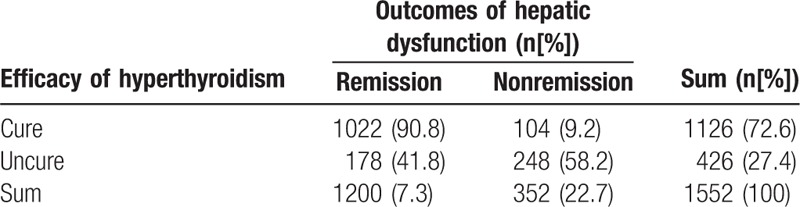
The relationship between efficacy of ^131^I treatment for Graves’ hyperthyroidism and outcomes of hepatic dysfunction after treatment.

The cure rate of Graves’ hyperthyroidism (including euthyroid and hypothyroidism) was 72.6% (1126/1552) after initial ^131^I therapy. Hypothyroidism occurred in about 34.1% of (530/1552) patients. The liver function tests in 90.8% (1022/1126) of the cured group patients returned to normal, although the remission rate of hepatic dysfunction was only 41.8% (178/426) in the uncured group of patients. The remission rate of hepatic dysfunction in the cured group was higher than that of the uncured group (*P* < 0.001). Thus, the prognosis for hepatic dysfunction was significantly associated with the outcomes of Graves’ hyperthyroidism.

## 4 Discussion

In clinical practice, hepatic dysfunction in Graves’ hyperthyroidism patients is common. Most of these patients have no obvious symptoms except for mild nonspecific discomfort. Only a minority of patients suffer from severe liver damage and develop liver failure. The mechanism may be related to the following factors:^[11–15]^ (1) long-term excessive thyroid hormones produnction has direct toxicity on the liver; (2) thyrotoxicosis hastens liver glycogen and protein decomposition, which causes liver cell degeneration; (3) A hypermetabolic state leads to an increasement in organic energy consumption and liver burden. However, the blood supply to the liver does not concomitantly increase. This causes hepatocellular anoxia and free radical damage; (4) autoimmune mechanisms might have a role in the process of hepatocyte injury; (5) hyperthyroid heart disease with cardiac insufficiency might cause hepatic ecchymosis and hepatocyte necrosis; 6. ATD has direct toxicity on the liver. It might be related to the individual's heterogeneous reaction.

Our study demonstrated that 65% of patients with Graves’ hyperthyroidism were associated with different degrees of hepatic dysfunction. Most of these patients had mild or moderate liver injury, and severe hepatic dysfunction occurred in only 6.6% of patients. Additionally, an increase in ALP was found to be the most common abnormal indicator, and the prevalence was 52.3%.

To explore the risk factors of hepatic dysfunction for patients with Graves’ hyperthyroidism, we analyzed 13 related factors, such as age and gender, and found that hepatic dysfunction is more likely to occur in patients aged ≥45 (OR = 2.386), a history of Graves’ hyperthyroidism >3 years (OR = 3.985), heart rate >90 bpm (OR = 1.617), FT_3_ level >19.5 pmol/L (OR = 2.116), TRAb concentration >15IU/L (OR = 1.857), and positive TPOAb (OR = 1.453) using univariate analysis (all *P* < 0.01). Additionally, we found that older subjects, cases with longer duration of Graves’ hyperthyroidism, higher FT_3_ level or higher TRAb concentrations were more likely to be associated with hepatic dysfunction as patients with Graves’ hyperthyroidism using multivariate logistic regression analysis. The findings are different from previous research data of Li's group.^[16]^ Li's research showed that the influential factors for hepatic function injury were age, hyperthyroidism duration, heart rate, thyroid weight, FT_4_, RAIU, TgAb, TPOAb, and TRAb. However, their research subjects were patients with hyperthyroid hepatic injury and those patients who had been treated with ATD were excluded from their analysis.

Older patients are more likely to have chronic cardiac insufficiency, hepatic vein congestion, or even liver centrilobular necrosis.^[17]^ The liver plays an important role in thyroid hormone metabolism. In those patients with long-term duration of Graves’ hyperthyroidism, excess thyroid hormones increase liver burden, and have direct toxicity on the liver, so the likelihood of hepatic dysfunction rises.^[12,14]^ Increased TRAb concentration indicates an active autoimmune response,^[18]^ which results in hyperfunction of the thyroid gland, an increased excitability of multiple systems and augmented metabolism.^[19,20]^ Our study showed that patients with hepatic dysfunction had higher TRAb levels, which corroborates recent reports.^[21]^ FT_3_ has more tissue-activity than FT_4_, and studies have confirmed excess T_3_ induces apoptosis of hepatocytes.^[22]^ Excessive thyroid hormones secretion may cause superfluous oxidative stress, an imbalance between pro-oxidant and antioxidant, which can then lead to hepatocyte damage.^[23]^ In addition, excessive thyroid hormones increase heart rate and cardiac output. However, in the liver, increased tissue oxygen consumption may cause hepatocellular anoxia resulting in liver injury.^[24,25]^ Thus, higher TRAb or FT_3_ level can increase the risk of hepatic dysfunction for patients with Graves’ hyperthyroidism.

In total, hepatic function indices returned to normal 6 months after ^131^I treatment in 77.3% of patients. Moreover, the remission rate of mild hepatic dysfunction (86.8%) was higher than that of moderate (65.5%) or severe dysfunction (55.9%), and the remission rate of hepatic dysfunction for hepatocellular injury type (91.7%) was higher than that for bile stasis (59.2%) or the mixed type (74.0%). Therefore, the remission rate of hepatic dysfunction is closely related to its severity and types. Additionally, our study revealed that progntosis of hepatic dysfunction was associated with the outcomes of Graves’ hyperthyroidism. The remission rate of hepatic dysfunction in the cured group of patients (90.8%) was higher than that in the uncured group of patients (41.8%). ^131^I treatment should be the prior choice for those patients with hepatic dysfunction in order to control hyperthyroidism timely and effectively.

The metabolic process of ^131^I usually does not cause radiation damage to the liver.^[26]^ In our study, hepatic dysfunction mildly deteriorated in the 2 weeks after ^131^I treatment in a small minority of patients with severe liver damage. This may be related to the early release of thyroid hormones and the transient aggravation of hyperthyroidism. We did not observe an obvious exacerbation of hepatic dysfunction through long-term follow-up.

Our study had some inherent limitations associated with the retrospective analysis. The 1552 patients involved in our study had hepatic dysfunction induced by Graves hyperthyroidism and/or ATD. Those patients with liver diseases (such as autoimmune liver disease) or biliary tract diseases were rejected. However, corresponding testing or examination was not universally performed to exclude these patients. Additionally, we selected the cases with complete data to perform our retrospective analysis. The exclusion of a few patients who were lost to follow-up might result in potential bias. Therefore, rigorous prospective studies will need to be performed to confirm these preliminary findings.

## 5 Conclusions

Hepatic dysfunction is more likely to occur in older patients, and cases with a longer history of Graves’ hyperthyroidism, and higher FT_3_ or TRAb concentrations. Additionally, the prognosis of hepatic dysfunction is closely associated with the outcomes of Graves hyperthyroidism after ^131^I treatment.

## Acknowledgment

The authors thank the native English speaking scientists of Elixigen Company (Huntington Beach, California) for editing our manuscript.
